# Applications of Microbiome Analyses in Alternative Poultry Broiler Production Systems

**DOI:** 10.3389/fvets.2019.00157

**Published:** 2019-05-24

**Authors:** Zhaohao Shi, Michael J. Rothrock Jr., Steven C. Ricke

**Affiliations:** ^1^Center for Food Safety, Food Science Department, University of Arkansas, Fayetteville, AR, United States; ^2^Egg Safety and Quality Research Unit, U.S. National Poultry Research Center, USDA-ARS, Athens, GA, United States

**Keywords:** pasture flock, microbiome, feed additive, preharvest, gastrointestinal tract

## Abstract

While most of the focus on poultry microbiome research has been directed toward conventional poultry production, there is increasing interest in characterizing microbial populations originating from alternative or non-conventional poultry production. This is in part due to the growing general popularity in locally produced foods and more specifically the attractiveness of free-range or pasture raised poultry. Most of the focus of microbiome characterization in pasture flock birds has been on live bird production, primarily on the gastrointestinal tract. Interest in environmental impacts on production responses and management strategies have been key factors for comparative microbiome studies. This has important ramifications since these birds are not only raised under different conditions, but the grower cycle can be longer and in some cases slower growing breeds used. The impact of different feed additives is also of interest with some microbiome-based studies having examined the effect of feeding these additives to birds grown under pasture flock conditions. In the future, microbiome research approaches offer unique opportunities to develop better live bird management strategies and design optimal feed additive approaches for pasture flock poultry production systems.

## Introduction

Pasture flock or free-range raised poultry continues to be a popular market option for retail poultry products for a variety of reasons including the attractiveness of being locally produced and sold for retail (1–4). As the production of naturally-raised poultry either as pasture flock or free-range grown chickens increases, consideration of factors such as environmental impact and food safety concerns have to be taken into account (4–6). This impact may include changes in nitrogen and phosphorus levels as well as antimicrobial runoff and pathogen contamination (6, 7). However, small poultry producer operations both in the U.S. and internationally are highly diverse in management styles and present challenges for making uniform recommendations (8, 9). Given this diverse range of management approaches, food safety problems can be somewhat unpredictable, and coupled with the more restrictive nature of mitigation options, present a challenge for restricting the prevalence of foodborne pathogens (7, 10). This combined with favorable public opinion regarding free-range livestock production, represents a dilemma for food safety risk management (11).

Foodborne pathogens that have been associated with free-range birds either in preharvest production or from retail birds include *Campylobacter, Listeria*, and *Salmonella* (12–19). Consequently, more focus is being directed toward developing acceptable methods for controlling and reducing the levels of prevalence of these pathogens on retail poultry products. One of the primary targets is live bird production where limiting foodborne pathogen establishment is certainly a driver but improving bird health and reducing mortalities are also important considerations. Along these lines, feed additives such as probiotics and prebiotics are attractive as they represent generally acceptable management practices and have been demonstrated to be at least somewhat effective in conventional poultry production (20–23). These issues have become more critical with the removal of antibiotics from conventional production. However, as with conventional live bird production, it has also become clear that designing optimal feed amendment approaches requires a better understanding of the avian gastrointestinal tract (GIT) system microbial ecology as well as the complexities associated with responses to the alteration of feed and feed amendments (24). With the emergence of sequence based assessment of microbial communities, it has now become possible to develop a much more comprehensive assessment of individual members of the microbial GIT communities. The objective of this review is to examine and discuss the use of these approaches for gaining a better understanding of the microbial populations in pasture or free-range poultry production.

## Pasture Flock Production– General Concepts

Poultry production in the early 20th century was historically characterized by small flock type farms with poultry viewed as supplemental income in a mixed food animal operation dependent upon multiple sources of revenue (3, 25–27). As commercial poultry production progressed over the 20th century and into the 21st century, the size of flocks increased dramatically with nutrition and breeding technologies advancing and the industry becoming vertically integrated to the point of large commercial flocks dominating the poultry meat market (25). However, a new market phenomenon has emerged in the past few years in the rise of locally grown free-range or pasture flock raised birds with on-farm processing (28, 29). While chicken can be marketed as either natural or organic, natural labeled poultry products generally outsell their organic counterparts (1).

Organically produced foods are much more rigorously regulated with requirements in place for all aspects of production and processing while natural poultry and other meats are only regulated from a post-processing side (1). Pasture raised poultry, when considered within a broad category, is defined as a production system where birds are raised outdoors in some sort of small, moveable, ventilated pen arrangement (16, 30, 31). Management of these pens in terms of frequency of rotation in a pasture, protection from predators, types of housing, and other requirements are described in detail by Fanatico (32). Housing can either be moveable or fixed with portable housing being easily moved without causing injury to the birds (16, 30). Given the diverse nature of these types of practices, several potential challenges exist for achieving consistent production levels to meet market demands over a period of time. Issues considered important for small producers may vary depending on the geographical region. For example, in a Minnesota-based survey, Jacob et al. (8) concluded that extension programs were needed by small scale antibiotic-free flock producers for feed and pasture choice, waste disposal, pre-slaughter feed withdrawal, and marketing. When Hilimire (33) surveyed California pasture flock growers, the primary issues identified by these farmers were predation of birds and feed costs.

On-farm poultry processing using mobile poultry processing units (MPPUs) has become an attractive means for pasture flock growers to process their birds in preparation for the market (29). The mobile characteristic of these processing units offer distinct advantages for rural small flock producers where the nearest processing facilities may be located at distances that preclude ready access (3). This is also consistent with the conclusion by Angioloni et al. (29) that MPPUs and on-farm processing setups cost more to purchase initially, but once established, enable a lower processing cost compared to off-farm alternatives. In addition, wastewater originating from on-farm processing and MPPUs generate lower total Kjeldahl nitrogen and total phosphate than conventional processing (6). While individual requirements for construction of MPPUs may vary from state to state, most are on some type of either open or enclosed wheeled trailer that houses most of the standard components of poultry processing, including kill cones, pickers, evisceration tables, chill tanks, and hand washing sinks (3). Similar to organic poultry production, the range of available sanitizers remains somewhat limited for pasture flock processing compared to conventionally produced poultry, and further research is needed to identify more antimicrobials that would not only be effective against foodborne pathogens but acceptable for MPPU application and economical for the producer (10, 34). This is due in part to the need to limit foodborne pathogen contamination originating from birds entering processing as well as from cross contamination events that may occur during processing (16).

## Pasture Flocks and Foodborne Pathogens

There are several unique and particularly challenging aspects of pasture flock poultry production including maintaining bird health, reducing mortalities, and limiting foodborne pathogen prevalence. Of the issues associated with pasture poultry flocks, foodborne pathogen occurrence has probably been the most extensively studied. Foodborne pathogens have been isolated from all phases of pasture poultry production from live bird production, during processing, and from birds marketed at retail. Most of the focus has been on *Salmonella* and *Campylobacter*, but other foodborne pathogens such as *Listeria* have been isolated as well. Milillo et al. (15) using an analytical profile index of *Listeria, sigB* allelic typing, and *hlyA* PCR tests found that both *Listeria monocytogenes* and *Listeria innocua*, including hemolytic *L. innocua*, could be isolated from the cecal and environmental (grass/soil) samples of pasture flock birds. Locatelli et al. (35) isolated *Listeria* spp. from fecal and soil samples in 15 % of the pasture fock poultry farms they surveyed and identified *L. innocua, L. monocytogenes*, and *L. welshimeri*.

Bailey and Cosby (12) isolated *Salmonella* from 31% of 135 free-range carcasses and 25% of 53 all-natural carcasses of birds that had not received meat, poultry meal, or antibiotics. Scheinberg et al. (36) saw similar prevalence for *Salmonella* when they examined whole chickens from farmers' markets and reported that 28 and 90% were positive for *Salmonella* spp. and *Campylobacter* spp., respectively. In a pasture flock surveillance study, Melendez et al. (14) identified 59 *Salmonella* isolates from pens, feed, water, and insect traps at the farm, as well as from retail carcasses obtained from a local natural foods store and a processing plant. When the *Salmonella* isolates were serotyped, the majority were *S*. Kentucky at 53%, *S*. Enteritidis (24%), *S*. Barelly (10%), *S*. Mbandaka (7%), *S*. Montevideo (5%), and *S*. Newport (2%). Interestingly, despite originating from antibiotic-free production systems, all isolates were resistant to sulfixasole and novobiocin, some were resistant to additional antibiotics, and most contained class I integrons. Additionally, a study by Rothrock et al. (37) found antibiotic resistance from poultry and environmental isolates of *E. coli, Salmonella, Campylobacter*, and *Listeria* from 15 all-natural, antibiotic-free, pasture flocks taken from six farms in the southeastern U.S. Using the NARMS antibiotic sensitivity protocols, Rothrock et al. (37) observed that levels of antibiotic resistance tended to remain consistent throughout the farm-to-fork chain. However, they also found that there appeared to be individual farm-level effects, shown by *Salmonella* only being isolated from three farms, and that high levels of antibiotic resistance in one genus did not correlate to resistance in others, highlighting the need to design specific assessments for resistance in public health studies. Other characteristics of foodborne pathogens may be influenced by conditions associated with free-range production systems. For example, Hanning et al. (13) characterized *Campylobacter* isolates from pasture flock farms, retail, and processing facilities over an 8 month time period and observed that the prevalence between conventional and pasture flock retail birds was similar. However, when they sequenced the short variable region of the *flaA* locus (*flaA* SVR) to genotype *C. jejuni* isolates, they noted that the genetic diversity of the *flaA* SVR genotypes increased from the farm to the carcass in pasture flock birds when compared with conventional poultry.

Some of the prevalence of foodborne pathogens in small-scale and pasture raised birds could be related to rearing conditions. Indeed, Lupatini et al. (38) demonstrated that organic farming increased taxonomic and phylogenetic richness, diversity, and heterogeneity of soil microbial consortia when compared to conventional farming. Tangkham et al. (39) tracked *Campylobacter* appearance on a weekly basis in eggshells, live birds, feed, and the drinking water in the rearing environments of small broiler operations where birds were raised either in open-air housing or environmentally controlled housing. They concluded that vertical transmission from eggs was not a factor but did note an increase in *Campylobacter* spp. in birds raised in open-air housing compared to those from environmentally-controlled housing. Li et al. (40) reported an increase in the incidence of *Salmonella* and *C. jejuni* recovered from the ceca of birds processed in a MPPU after being raised on built-up litter when compared to birds reared on clean shavings. The method of processing may not be a factor. Trimble et al. (17) compared pasture raised broilers processed on farm sites, a small U.S. Dept. of Agriculture inspected facility, and a MPPU pilot plant facility and concluded that birds generally were contaminated with *Salmonella* and/or *Campylobacter* regardless of the type of facility. However, Li et al. (40) reported that post-chill application of antimicrobials can successfully reduce *Campylobacter* and *Salmonella* carcass contamination. Reduction of foodborne pathogens during the processing of pasture flock birds may be important, not only for decreasing levels on the retail birds but also for reducing pathogen loads in the processing waste disposal onto the soil (18).

## Microbial Ecology in Pasture Flock Poultry– General Concepts

As pasture flock poultry production continues to grow to meet market demand, increased efforts will be needed to develop systematic approaches to improve the microbial safety of the product during all phases of the farm-to-fork continuum. This remains a challenge due to the diversity in locations of farms and management practices. This is important because part of the development for more optimal antimicrobials as well as control measures during the grow-out of pasture flock birds requires an understanding of the microbial ecology not only of the GIT in the bird, which can harbor high levels of foodborne pathogens, but also during processing of the bird into meat products. Non-pathogenic indigenous microbial communities in the avian GIT can influence the ability of the respective pathogen to colonize and become established (41). Feed amendments such as probiotics and prebiotics can alter or shift the GIT microbial population to become more of a barrier to pathogen colonization while other agents such as antimicrobial chemicals and biological agents such as bacteriophages can decrease pathogen populations already established (42–44). The concern during processing is not just the presence of pathogens but the general bacterial load that, depending upon the quantity and type of organisms present, can decrease retail shelf life of the processed bird. In short, better characterization of the microbial populations is needed to develop a comprehensive approach to targeting microbial populations present in these different phases of pasture flock production.

Classic culture-based microbiology has been insightful for some aspects of microbial ecology in food animal systems. However, limitations in the recovery of representative viable organisms may lead to under representing certain microbial populations such as with strict anaerobes in the GIT. This has resulted in an incomplete picture of the impact of feed amendments such as probiotics (45). More recently, next generation sequencing (NGS) technologies have become routine and the opportunity to characterize a microbial population in its entirety without relying on culturing is now possible. Based on the sequencing of the 16S rRNA gene, microbial taxa can be identified as function of operational taxonomic units (OTUs) and differences in the microbial community from independent samples or sampling sites can be compared as a function of microbiome composition (46). Improvements in computer program pipelines and bioinformatic tools offers in-depth analyses of microbiomes and delineation of specific factors that may be influential on overall microbial communities as well as individual members of the respective communities and potential integrative networks among groups of organisms (46–48). Microbiome analysis techniques have certainly been used extensively to study poultry GIT responses to different treatments and to a lesser degree poultry processing microbial populations (49, 50). However, much less research has been done on microbiome analyses with pasture flock birds even though the differences in the microbial communities would likely offer an opportunity to delineate specific patterns based on the respective microbial consortia profiles and potentially predict outcomes in response to changes such as general dietary modification or inclusion of specific feed additives. In the following sections the microbiome research that has been done with pasture flock poultry will be discussed.

## Potential Factors that Influence GIT Microbiomes in Pasture Flock Birds

Based on conventional poultry production studies, the GIT microbiome composition and intestinal function of the bird is influenced by several factors, some more obvious than others. Factors which impact the diversity of the microbiota in the bird and GIT function can originate from diet, stocking density, geographical location, feed additives such as, bird age, bird environment, and pathogen presence among other less well defined factors (51–56). Given the diverse nature of pastures with potential differences in forages as well as exposure to a wide range of environmental conditions, it would not be surprising that the birds' GIT microbial populations might also reflect this diversity. Likewise, differences in the length of growth cycles, utilization of slower growing bird breeds, stocking density, and potential contact with wildlife could be influential as well.

While these factors may potentially influence the microbiome diversity in pasture raised birds, only minimal research has been conducted with birds from these types of production systems. The majority of poultry GIT microbiome work has focused on birds raised under conventional management practices and any conclusions pertaining to pasture flock birds need to be extrapolated from the outcome of these studies. Some factors such as exposure to wildlife would be considerably different than conventional poultry production systems but much less is known on wildlife microbiomes. Hird (57) has pointed out that captivity alters the microbiome and that the birds yield highly diverse microbiomes. However, some of the work that has been done with wild bird species may have potential relevance. For example, when Bodawatta et al. (58) sequenced the GIT of New Guinean passerine bird species, they noted a dietary influence with more microbial diversity detected in the omnivore species than in the insectivore species, with insectivore GITs consisting mainly of lactic acid bacteria. Since pasture flock birds would have access to a variety of insects, it would be of interest to compare their GIT taxonomy with wild birds that consume insects as a proportion of their diet. Teyssier et al. (59) observed that during the later stages of nestling development of the Great Tit (*Parus major*) passerine bird, the nest environment impacted the composition of the GIT. Along these lines, in the domesticated Peking duck, Best et al. (60) demonstrated that GIT populations were different in aviary-raised ducks vs. barn-raised birds.

Diet differences between conventionally raised poultry and pasture flock birds may also be a distinguishing influence on the GIT microbiota, particularly if low nutrient diets are used to ensure slower growth in pasture flock birds (61). Even when diets are quite similar, differences between GIT microbiota may still be observed. For example, when de Greeff et al. (62) compared jejunal gene expression in layer hens fed either conventional or organic diets of an otherwise identical composition, they detected differences in the expression of 49 genes, including those associated with cholesterol synthesis and immunological processes. In addition, pasture flock birds have access to a much wider variety of food sources such as insects and forages in addition to the formulated diets provided. Whether fiber intake occurs from the forages present on pasture presents another unknown. Low fiber diets have been shown to alter gizzard function and have been touted as a means to maintain proper GIT function and improve overall bird performance (63, 64). There is likely an impact on the GIT microbiota as well. The cecal microbiota of layer hens and chicks have been demonstrated to be capable of fermenting fiber sources such as alfalfa, causing subsequent effects on the GIT microbiota (65–70).

Age and breed of bird are likely factors as well. Kers et al. (71) concluded that host related factors of sex, age and breed exhibited considerable impact on GIT microbial populations with differences in microbial community composition between layer and meat-type chickens. Lumpkins et al. (72) compared Athens Canadian Random Bred (ACR) broilers with modern multipurpose bird strains and high yield bird strains and detected differences in bird performance, GIT measurements, and the GIT microbial consortia between the ACR birds and the modern bird strains. It is likely that these effects may also be observed in pasture flock raised birds as well. For example, Hanning et al. (73) observed differences in body weight responses to fiber or prebiotic supplemented diets between Naked Neck slow-growing birds vs. Cornish White Rock cross fast-growing broilers reared under pasture flock conditions. Age and development of the avian GIT also appears to greatly influence GIT microbiome composition. *In vitro* cecal incubations using inocula sourced from birds of different ages support the impact of age on microbiome composition and ability to inhibit *Salmonella* introduced into the incubation (74, 75). Future studies will need to be conducted specifically with pasture flock birds to delineate the relative levels of influence that age vs. breed have on the development of the GIT microbiota to establish a baseline for additional comparisons with variables such as impact of diet and environment.

## Feed Additives and Pasture Flock GIT Microbiomes

Given the environmental stresses and other challenges associated with pasture flock poultry production, choices in feed additives and dietary modulators are an important consideration to improve bird health, reduce mortalities, and limit foodborne pathogen establishment. Several feed additives have been suggested over the years that could potentially be used in pasture flock and/or organically raised poultry and replace antibiotics in conventional poultry production. These include bacteriophages, botanical products, organic acids, probiotics and prebiotics, and others (10, 22, 34, 76–82). Most of these feed additives have only been suggested as potential agents for use in alternative poultry production and have only had minimal research conducted with pasture flock poultry operations. However, some pasture flock research has been conducted with prebiotic supplementation that determined GIT microbial population responses.

Prebiotics are compounds, usually complex carbohydrates, which cannot be directly utilized but can be fermented by GIT bacteria, particularly members that are considered beneficial to the host such as bifidobacteria and lactic acid bacteria (21–23, 78, 83–85). Considerable emphasis has been placed on prebiotics as one of the candidates to replace antibiotics in conventional poultry production but there have only been a few isolated studies on pasture flock poultry (22, 23). Initial research conducted on pasture flock poultry and prebiotics focused primarily on the supplementation of commercial probiotics and their impact on bird performance and meat quality characteristics. After feeding probiotics and prebiotics from bacterial and yeast sources to free-range broilers, Pelícia et al. (86) reported lower mortalities and greater weight gain in birds fed the bacterial-based prebiotic, while both bacterial and yeast-based probiotics and prebiotics improved carcass yield when compared to control birds. However, some of the responses may be poultry breed dependent as well as specific for a particular type of prebiotic or dietary supplement. Hanning et al. (73) reported that free-range raised fast-growing Cornish Cross White Plymoth Rock broilers fed diets supplemented with the prebiotic fructooligosaccharide (FOS) exhibited a higher final body weight (8 weeks) while slow-growing Naked Neck free-range birds on a fiber source (plum fiber) had greater final body weight gains.

With advances in molecular techniques for microbial identification and characterization, more recent pasture flock studies have included in-depth analyses of the GIT microbiota. Park et al. (87) examined the response of Naked Neck chicks fed commercial yeast-based prebiotics while being raised in pasture pens that were moved twice a week. They did not detect differences in feed conversion ratios, live bird body weights, or post-processing body weights, however the commercial prebiotics did decrease cecal *Campylobacter* populations. Using a PCR-based denatured gradient gel electrophoresis (DGGE) method to compare cecal microbial populations from birds fed different commercial prebiotic treatments, Park et al. (87) also found that the prebiotic cecal populations were more related within their respective groups than control bird cecal populations. When individual bands were excised from the DGGE bands and sequenced, *Bacteroides slaanitronis* was identified in all treatment groups while *Barnesiella ciscericola* and *Firmicutes* were detected only in the prebiotic treatment group ceca. The authors concluded that DGGE could be useful in easily detecting shifts in cecal populations from prebiotic usage despite the limitations in the technique.

Development of NGS techniques for routine microbial population characterization based on 16S rRNA gene comparisons have greatly improved the ability to conduct comprehensive in-depth GIT microbiome analyses (24, 46). Park et al. (88) used an Illumina MiSeq platform based on the V4 region of the 16S rRNA gene to identify cecal populations in free-range birds fed two commercial yeast cell wall-based prebiotic compounds. Diversity differences among the treatments were relatively minimal with the two products resulting in different levels of OTUs, one similar to that of control birds and the other yielding lower numbers of OTUs. When microbial population diversities were compared among the groups, the two prebiotic fed groups and the control group cecal populations were distinctly clustered on unweighted principal coordinated analysis (PCoA) Unifrac plots. Taxonomic analyses revealed a somewhat minimal impact by both prebiotics at the phyla level, although one of the yeast prebiotics did lead to an increase in *Proteobacteria* and *Cyanobacteria* OTUs compared to the other treatments while increased OTUs of *Firmicutes* were detected in the control diet fed bird ceca. At the genus level, one of the yeast cell prebiotics led to an increase in *Faecalibacterium*. Overall it appeared that microbiome analyses could successfully detect differences in cecal microbial populations from pasture flock poultry fed different prebiotic containing diets even when the prebiotic sources were derived from similar commercial sources.

The impact of age on microbiome population composition could also be shown through 16S rDNA analysis. Park et al. (89) utilized microbiome sequencing to compare cecal microbiota populations from chickens with plum fibers, FOS, or GOS feed additives and found cecal populations to be impacted by their respective treatments. As the plum fiber and FOS fed birds aged (2–6 weeks), Shannon diversity indices increased while the total number of OTUs did not increase appreciably for control birds. However, when phylogenetic clustering for each treatment was compared, bird age had a much greater impact on clustering patterns than that from the corresponding treatments. Analysis based on correlations with metadata found that host age and developmental stages were the key contributors to microbial community diversity. The influence of age on GIT microbial composition has also been reported by Cui et al. (90) in young vs. older hens in both caged and free-range birds. Further analyses breakdown of OTUs revealed that the genus *Alistipes* increased with age across all bird groups and could be a potential predictive indicator for age, weight, and *Campylobacter* populations. *Lactobacillus intestinalis* was also predictive for *Campylobacter* as well as the presence of FOS, GOS, and plum fiber in birds at 2 weeks of age. Clearly age is a major driver of changes in microbial diversity to the point of masking other factors; however, this could be different depending on the stage of bird development. It would be interesting to examine the changes in diversity during the first 2 weeks of age in birds when different dietary amendments are introduced to determine whether age is still a predominant factor.

## Future Directions

As pasture flock poultry markets continue to grow in popularity, there will be an increasing need to develop systematic approaches for optimizing management practices to reduce mortalities, improve health, and limit pathogens. The introduction of feed additives that are considered acceptable by the both producers and consumers offers a means to potentially achieve some of these goals. However, more research is needed to assess and develop consistent baseline patterns of production responses that could be viewed as some sort of standard for evaluating newly developed feed additives. To accomplish this, factors such as breed differences, environmental impact, and dietary management would have to be considered. As a part of this evaluation, the impact on bird responses due to changes in the GIT microbiota is emerging as an important factor as well. With the introduction of more advanced and cheaper sequencing methods, in-depth assessment of GIT microbial responses has now become a reality. In the limited set of studies conducted thus far, it appears that age is one of the more important factors impacting microbiome diversity development. Whether dietary amendments such as prebiotics can also have an impact will need additional studies with a broader spectrum of prebiotic compounds and more frequent incremental sampling to delineate age vs. treatment influence on the microbiome.

There are other opportunities to apply microbiome analyses to pasture flock poultry production operations. While there has been a focus on studying the pasture flock birds, much less has been done to determine the impact of these free-range birds on their surrounding environments. Rothrock et al. (37) found that the antibiotic profiles of soil samples exhibited similar rates of antibiotic resistance as that from fecal samples from pasture flocks of birds, demonstrating the potential impact they may have. Public health studies, particularly those that focus on antimicrobial levels, must account for the role of poultry flocks on the environment (91). This could be a critical issue to consider given the placement of pens in pastures and the exposure of the soil and fresh water sources to these flocks with microbial populations shared by pasture flock birds potentially influenced by the presence of these birds. Whether the density of pasture flock birds would be sufficient to produce similar alterations in the soil microbiota remains to be determined, but it is conceivable that the frequency of moving pens vs. remaining in one place for an extended period of time may impact the soil in proximity of the pen.

Microbiome analyses could potentially also be informative for the assessment of pasture flock poultry processing. Deciding optimal sanitizers and antimicrobials is dependent on microbial profiling, usually done with combinations of non-selective and selective culture plating to enumerate the respective spoilage and foodborne pathogen microbial populations. Microbiome mapping has been done with conventional poultry processing and proven to be useful for following shifts in microbial populations during the various processing stages (50, 92). In the course of conducting the sequencing analyses and taxa identification, potential indicator microorganisms have been identified that offer predictable baselines for intervention evaluations. It is anticipated that microbiome approaches could be applied to pasture flock processing to achieve similar outcomes.

Improvements in sequencing technologies such as further development of long read sequencing platforms such as the Oxford Nanopore and Pacific Bioscience sequencers along with further development of fourth generation sequencing technologies offer opportunities for deep sequencing of microbial communities both in the GIT as well as in poultry processing microbial communities (93). Along with improved sequencing resolution data analyses will become more sophisticated with advanced statistical power to achieve correlations and network construction of the microbial communities and elucidate host genome wide-microbiome relationships (94). This could prove to be particularly important with the diverse chicken breeds used in pasture flock operations. Likewise, identification of non-pathogenic indicator organisms reflective of pathogens and other factors both in live bird production and processing may be more likely. This would allow for improved prediction of feed additive and antimicrobial strategies. Finally, as sequencing technologies and bioinformatics become more advanced it may become possible to link microbial population patterns back to the live bird flock prior to slaughtering and use this information to optimize sanitizer applications to retain fresh product shelf life.

## Author Contributions

ZS, MR, and SR wrote and edited the manuscript. All authors significantly contributed to the work of this review.

### Conflict of Interest Statement

The authors declare that the research was conducted in the absence of any commercial or financial relationships that could be construed as a potential conflict of interest.
